# Advancing TMS for PTSD – From networks to individuals

**DOI:** 10.1016/j.transm.2025.100178

**Published:** 2025-06-30

**Authors:** Patlapa Sompolpong, Sanne J.H. van Rooij

**Affiliations:** Graduate Division of Biological and Biomedical Sciences, Emory University, United States; Department of Psychiatry and Behavioral Sciences, Emory University School of Medicine, United States

**Keywords:** Posttraumatic stress disorder (PTSD), Personalized targeting, Resting state functional connectivity (rs-FC), Lesion network mapping, Threat neurocircuitry, Amygdala, Neuroimaging

## Letter to the editor

In this letter, we summarize the current gaps and limitations in using Transcranial Magnetic Stimulation (TMS) to treat posttraumatic stress disorder (PTSD), while highlighting the potential of connectivity-guided TMS to more precisely target the neural circuits involved in aberrant fear learning and threat detection - core features of PTSD.

The first line of treatment for post-traumatic stress disorder (PTSD) is trauma-focused psychotherapy to extinguish traumatic memories, sometimes with pharmacological treatments. While effective for most patients, a notable 30–50 % do not respond to current treatment options, signifying the need for alternative approaches. Such variance in efficacy may be due to heterogeneity in PTSD manifestation (Galatzer-Levy & Bryant, 2013) or individual differences in neurobiological profiles that may hinder extinction processes and predict treatment non-response (van Rooij et al., 2021). Leveraging increased knowledge of neurobiological signatures of PTSD, a novel direction in the field is to develop individualized treatments that consider each patient’s unique brain connectivity and result in improved clinical efficacy.

Transcranial magnetic stimulation (TMS) has emerged as a noninvasive intervention that has been FDA-approved for several conditions including treatment-resistant major depressive disorder (MDD). TMS is a promising treatment modality for PTSD, possibly offering greater tolerability over trauma-focused therapies that may be difficult for patients to complete and benefit from due to its emotionally-triggering nature. Thus far, studies using TMS for PTSD have adopted targets and parameters from depression research. While these studies targeting dorsolateral prefrontal cortex (DLPFC) have shown moderate success (reviewed in e.g., Petrosino et al., 2021; Brown et al., 2025), response rates are highly variable (29–63 %), and both treatment efficacy and duration could likely be improved by a mechanistic understanding of TMS for PTSD. Previous TMS trials for PTSD (with comorbid MDD) collected baseline magnetic resonance imaging (MRI) scans to assess mechanisms and identified neuroimaging predictors for clinical improvement (Philip et al., 2018; 2019), including greater ventromedial (vm)PFC-amygdala functional connectivity (FC) (Philip et al., 2018). Treatment non-response in PTSD is predicted by exaggerated threat neurocircuitry responses to emotional cues, most notably the amygdala, which is postulated to prevent extinction of traumatic memories (first shown in Bryant et al., 2008). Based on decades of multidisciplinary research consistently showing fear-learning and threat-detection circuits to predict PTSD development and maintenance (e.g., Fenster et al., 2018), TMS targeting these circuits may be promising in treating PTSD (van Rooij et al., 2021; Brown et al., 2025).

There are numerous elements of TMS to consider, including stimulation frequencies, treatment sessions and dose, as well as the context in which TMS is delivered (e.g., van Rooij et al., 2024a). Extensive research is ongoing to determine the impact of different parameters on efficacy for different disorders. The core element most directly influenced by advancing knowledge of neurobiology underlying psychiatric disorders is the target location. Scalp-based heuristics have traditionally been used to locate an anatomical target but fail to account for interindividual neuroanatomical differences. Variability in targeted sites across individuals may engage distinct functional networks and contribute to variation in clinical outcomes (Opitz et al., 2016). More recently, structural MRI has been incorporated, and neurocircuitry-based targets have been investigated. Although it remains to be confirmed if neuronavigation methods provide clinical superiority over conventional targeting, it allows for more reliable targeting across time and generates highly robust and reproducible targets for a given individual (Cash & Zalesky, 2024).

Research in depression has led the way for defining targets based on neural circuitry instead of isolated regions. One such approach is lesion network mapping (LNM), in which lesion locations associated with specific symptoms in different patients are mapped onto a standard atlas to define networks causally related to depression (Padmanabhan et al., 2019). Distinct regions implicated in discrete symptom clusters have been identified using LNM, and these structural targets can now be used to guide targeting to treat specific symptoms (Siddiqi et al., 2020; Taylor et al., 2024). This group-level method has provided interesting new targets for several disorders and symptoms (e.g., Joutsa et al., 2022; Pines et al., 2025), however, it does not account for interindividual differences in FC that may dictate clinical outcomes (Fox et al., 2013).

A second approach aims to modulate the circuitry known to be implicated in the disorder by using resting state functional connectivity (rs-FC) to guide targeting (Fox et al., 2014). Previous work found that DLPFC stimulation sites more strongly connected to the subgenual anterior cingulate cortex (sgACC), a structure long implicated in depression, were associated with better clinical outcomes (Fox et al., 2012; Weigand et al., 2018). This line of research suggests targeting based on connectivity with subcortical structures or associated networks affected in depression may improve TMS outcomes. This led to development of the first use of fMRI in an FDA-approved TMS protocol for depression using individualized targets (Cole et al., 2020, 2022). Outcomes were significantly better when stimulation sites were closer in proximity to the individualized target than a group-derived canonical target, highlighting the clinical potential of individualized targeting (Cash et al., 2021). A recent trial in patients with comorbid PTSD/MDD also found preliminary evidence that rs-FC-guided targeting is superior to scalp-based targeting in improving symptoms at a long-term (6-months) follow-up (Oathes et al., 2024, preprint), suggesting the potential advantage of personalized targets for long lasting clinical improvements.

Circuit-based and neurotargeting approaches have been applied to and are being further developed for PTSD. First, following their groundbreaking work in depression and other disorders, Siddiqi et al. (2024) recently defined a PTSD circuit that could potentially serve to guide targeting using LNM. They propose that this circuit is: 1) driven by FC between regions including medial (m)PFC and amygdala, regions previously implicated in PTSD across preclinical, human neuroimaging and lesion studies, and 2) generalizable beyond lesions, specifically FC within the circuit may guide optimal TMS targeting. Group-level FC mapping suggests the mPFC, which has the peak positive connection to the circuit, as an optimal target to facilitate extinction. The mPFC appears to be a promising target and is believed to be a critical inhibitory region associated with impaired fear inhibition and failure to promote extinction in a safe context in PTSD (van Rooij & Jovanovic, 2019).

The vmPFC has been the focus of prior neuromodulation treatments (Isserles et al., 2021; van ‘t Wout-Frank et al., 2024) though outcomes have varied, with one TMS study showing worsening of symptoms. A potential reason for variability is the context in which stimulation is delivered (i.e., state-dependency), with Isserles et al. activating the trauma before delivering TMS thereby inadvertently interfering the extinction process, compared to van ‘t Wout-Frank et al. who delivered tDCS during exposure thereby improving safety learning and reconsolidation. Notably, functional neuroimaging studies also show mixed results for the vmPFC, as both greater and lower vmPFC reactivity predict treatment outcome depending on the specifics of the task (reviewed in van Rooij et al., 2021). Finally, the structural boundaries used to define the vmPFC vary across research studies, which further emphasizes the need for specificity. Instead of proposing the mPFC as a universal target, Siddiqi et al. combined individual-specific rs-FC with their circuit to individualize targeting and confirmed clinical efficacy in one patient. They propose that stimulating prefrontal targets functionally connected to their PTSD circuit may trigger network-level changes that drive clinical improvements. However, the mechanisms and neurobiological premise of modulating an entire PTSD circuit instead of one region needs further consideration. Ultimately, a randomized trial is critical to determining mechanisms, clinical value, and feasibility of targeting this circuit.

A second novel approach for PTSD uses rs-FC to target subcortical regions within a network. Although TMS can only directly modulate cortical areas, accumulating evidence shows downstream effects on subcortical areas, notably TMS to the PFC modulates amygdala activity (Sydnor et al., 2022). Given strong evidence of heightened right amygdala reactivity as the primary driver of PTSD and hyperarousal (e.g., Stevens et al., 2013, 2017), connectivity-guided targeting can be used to identify cortical targets aiming to indirectly modulate the right amygdala. The DLPFC has reciprocal connections to the amygdala (Comte et al., 2016) and provides a good point of access for TMS. The premise is that low-frequency stimulation to right DLPFC will indirectly dampen amygdala hyperactivation, thereby facilitating extinction processes that may become altered with hyperarousal and resulting in overall symptom reduction. Right DLPFC stimulation has been shown to be feasible and effective for PTSD, but the optimal location for functional connectivity with the amygdala differs by individual (van Rooij et al., 2024b). In the first FC-guided personalized TMS clinical trial for PTSD, we defined individual targets within the right DLPFC with greatest FC with the right amygdala, and tested effects on PTSD symptoms and biomarkers in a randomized double-blind clinical trial for PTSD (https://www.clinicaltrials.gov/study/NCT04563078), specifically right amygdala threat reactivity, functional connectivity, and sympathetic arousal. Results from the study will provide important information on the effects of personalized TMS on PTSD symptoms and modulation of the threat neurocircuitry. Furthermore, pre- and post-TMS assessments of FC will contribute to advancing personalized neuromodulation for PTSD.

This is an exciting time for personalized neurotherapeutics for PTSD. While different approaches and specific targets have recently emerged, they seem to converge on the same neural circuit, which was eloquently validated in the study by Siddiqi et al. (2024). This circuit is also supported by two prior TMS studies for PTSD (and MDD) demonstrating pre-TMS rs-FC within and between large-scale networks to predict clinical changes including vmPFC-amygdala rs-FC (Philip et al., 2018, 2019). While Philip et al. (2019) did not prospectively define targets using rs-FC, they chose the right DLPFC under the premise it could lower amygdala threat reactivity, and scalp-based targeting may have worked under similar mechanisms as proposed by van Rooij et al. (2024b). Depending on the networks engaged with left DLPFC stimulation, clinical benefit may result from engaging the large-scale mechanisms as proposed by Siddiqi or networks often targeted for MDD, which may be effective due to high comorbidity of MDD in PTSD patients. Future work needs to determine whether targeting based on connectivity within a group-level circuit or connectivity to a subcortical region of interest are both effective and feasible approaches for treating PTSD with TMS and superior to scalp-based targets. It is possible that one approach may be clinically superior for certain symptoms. For example, indirect inhibitory stimulation of the amygdala may be more effective in dampening hyperarousal and associated symptoms suggesting a bottom-up approach, while excitatory stimulation of prefrontal areas with peak connectivity to a PTSD circuit may be more successful in promoting emotional regulation commonly impaired in PTSD through top-down learning (Liberzon & Sripada, 2007). Mechanistic neuroimaging studies are needed to determine direct effects of stimulation on the neurocircuitry. Other important considerations include treatment feasibility and tolerability by patients, as well scalability of individualization given the need for MRI scans to map targets. While identifying optimal treatment parameters requires further thorough investigation, the field is moving rapidly to advance treatment options for the many individuals suffering from debilitating symptoms of PTSD.
